# The Radial Arc technique: A systematic ultrasound method to imaging the distal biceps brachii tendon from a medial approach with anatomical insights

**DOI:** 10.1177/1742271X251337389

**Published:** 2025-05-13

**Authors:** Stavros Daoukas, Dimitrios Galanis

**Affiliations:** 1 Institute of Health and Social Care, School of Allied and Community Health, London South Bank University (LSBU), London, UK; 2 Radiology department, Queen’s Hospital NHS Trust, Barking Havering and Redbridge University Hospital (BHRUT), London, UK; 3 12th Orthopaedic Department, Metropolitan General Hospital, Athens, Greece; 4 SonoPedics, London, UK

**Keywords:** Point-of-care-ultrasound, tendon pathology, elbow anatomy, sonographic technique, ultrasound imaging

## Abstract

**Introduction:**

Distal biceps brachii tendon disorders predominantly result from repetitive use and eccentric loading. High-resolution ultrasound has emerged as a preferred diagnostic tool due to its dynamic imaging capabilities, accessibility, and cost-effectiveness.

**Topic Description:**

This paper introduces the Radial arc technique, a didactic and systematic ultrasound scanning method from a medial approach designed to facilitate the identification of the distal biceps brachii tendon insertion in the long-axis view. The technique is a five-step sonographic approach navigating sonographers and clinicians through a series of landmarks to address the often-complex sonographic examination of the distal biceps brachii tendon insertion. The paper also provides detailed anatomical insights into the biceps brachii muscle and the distal tendinous complex, highlighting key morphological features critical for accurate ultrasound interpretation.

**Discussion:**

The proposed approach is tailored to support the educational development of undergraduate and postgraduate students specialising in musculoskeletal sonography and enhance the practical skills of early-career and experienced sonographers and clinicians utilising point-of-care ultrasound. The anatomical framework provided offers a deeper understanding of the distal biceps brachii tendinous complex, supporting diagnostic accuracy for distal biceps brachii tendon-related conditions that are critical for guiding patient management.

**Conclusion:**

The systematic nature of the Radial arc technique aims to standardise practices not only in clinical settings but also in the design and execution of research studies involving the assessment of the distal biceps brachii tendon integrity with ultrasound. Future research should focus on assessing the reproducibility of the Radial arc technique in diverse clinical settings and among different practitioners and operators, crucial for its adoption in sonographic diagnostics.

## Introduction

Distal biceps brachii tendon (DBBT) disorders most often affect the dominant arms of middle-aged males and generally result from repetitive use and/or eccentric loading during active elbow flexion.^1,2,3^ Although tendinous ruptures are relatively uncommon, with an estimated incidence of 1.2–9.6 per 100,000 individuals annually,^4^ other pathologies, such as tendinopathic changes, bicipitoradial bursitis and other space-occupying lesions, can also affect the DBBT. Accurate diagnosis is therefore essential to inform patient management. In the context of complete ruptures, timely surgical intervention is critical to restoring the tendon’s function and preventing significant declines in supination force, with flexion force affected to a lesser degree due to compensation from the brachialis muscle.^5,6^ While magnetic resonance imaging (MRI) remains the gold standard for diagnosing such injuries, its correlation with surgical findings in cases of partial tears has not always been consistent.^7^ Consequently, high-resolution ultrasound has emerged as a preferred alternative due to its dynamic imaging capability,^8^ immediate availability,^9^ and cost-effectiveness,^10^ factors critical for early diagnosis and management of a range of DBBT pathologies.

Despite these advantages, the ultrasound examination of the DBBT is recognised for its complexity, often characterised as not straightforward and regarded as challenging.^7^ Various approaches, including the anterior, posterior, lateral, and medial, have been explored to optimise visualisation of the DBBT. Among these, the medial approach has been shown to be the preferred method offering superior visualisation with the longest average visualised tendon length at 4.8 cm and allowing complete visibility of the tendon and insertion during dynamic assessment, unlike the other approaches.^1^ However, achieving consistent results on ultrasound demands a high degree of precision and expertise.^10^

Considering these challenges, a more structured and didactic method for conducting ultrasound sonographic assessments of the DBBT is warranted. Although the medial approach, through the pronator teres window, is widely used for its superior visualisation and diagnostic accuracy,^1^ no systematic, step-by-step guide currently exists to simplify this technique. Hence, the aim of this paper is to propose a new systematic scanning technique, the ‘Radial arc’ technique, to facilitate the identification and visualisation of the DBBT in an accessible, easy-to-follow manner. Standardising this approach aims to democratise the skill, thereby making it accessible to a broader range of clinicians and thereby improving diagnostic outcomes across diverse clinical settings.

## Anatomy of the biceps brachii muscle and distal tendinous complex

### Muscle anatomy

The biceps brachii muscle (BBm), which encompasses both the long (LHBm) and short heads (SHBm), exhibits remarkable morphological diversity which bears significant clinical and functional implications. While the two-headed structure is most common, observed in 59% of cases, additional heads – typically a third – are present in 4.2% to 19.8% of individuals.^11^ While it is rare for either the LHBm or SHBm to be completely absent, these supernumerary heads, which may originate from locations such as the humerus, coracoid process, pectoralis major muscle, or humeral joint capsule, can pose clinical challenges due to their proximity to neurovascular structures, including the brachial artery and musculocutaneous nerve. This anatomical diversity can result in complications like neurovascular compression, underscoring the clinical relevance of these variations.^11^

Although literature on the ultrasound appearance of these supernumerary heads is limited, clinicians should remain aware of these potential variants, as they may deviate from the typical two-headed structure of the BBm and impact routine ultrasound assessments, particularly in differential diagnoses involving neurovascular compression.

### Internal and external bicipital aponeuroses

**Figure 1.**
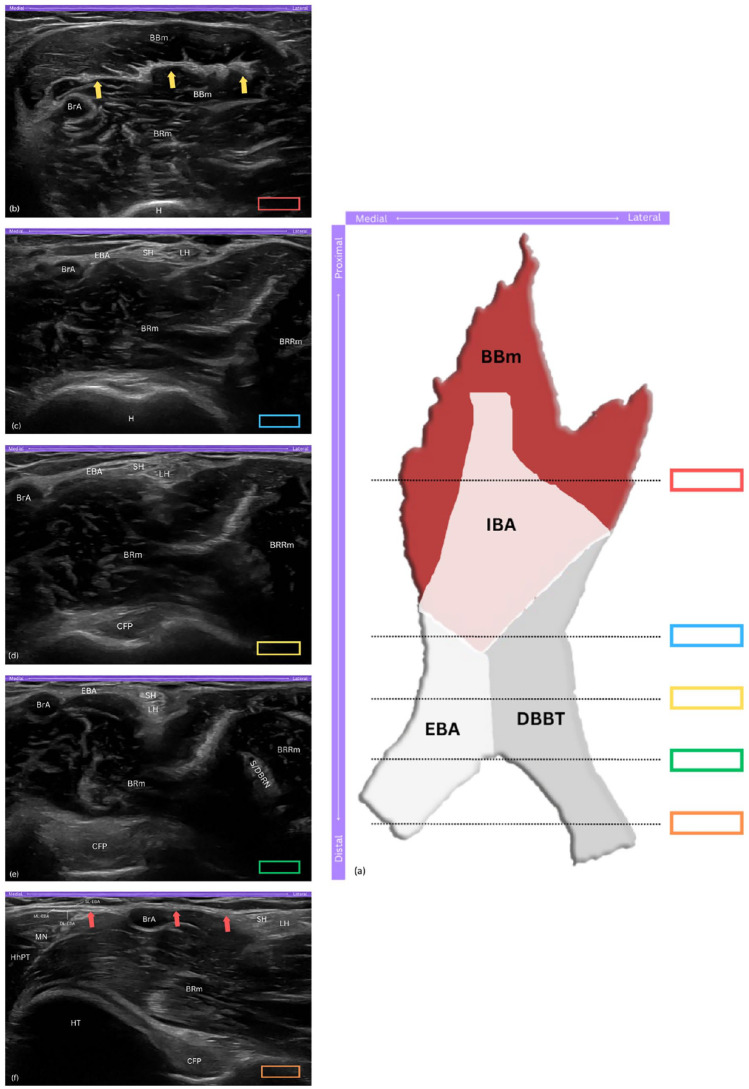
The distal biceps brachii tendinous complex (DBBTC). (a) Schematic drawing of the DBBTC components from the anterior aspect of the elbow, with coloured rectangles indicating probe positions corresponding to ultrasound views in panels (b)-(f). (b) Short-axis view of the hyperechoic, flat appearance of the internal bicipital aponeurosis (IBA) within the biceps brachii muscle (BBm). (c) Short-axis view showing the short head (SH) and long head (LH) of biceps brachii tendon, and external bicipital aponeurosis (EBA) arising from the IBA. (d) Short-axis view of the SH and LH approximating as they descend distally. (e) Short-axis view showing the 90° rotation, with the SH sliding to a superficial position over the LH. (f) Long-axis view of the EBA, travelling medially and superficial to the brachial artery (BrA), and merging with the ulnar flexor muscles; note the distinct superficial, middle, and deep layers of the EBA. DBBTC, distal biceps brachii tendinous complex; IBA, internal bicipital aponeurosis; EBA, external bicipital aponeurosis; DBBT, distal biceps brachii tendon; BBm, biceps brachii muscle; BRm, brachialis muscle; BrA, brachial artery; H, humerus; yellow arrows, IBA; BRRm, brachioradialis muscle; SH, short head of biceps tendon; LH, long head of biceps tendon; CFP, coronoid fat pad; S/DBRN, superficial and deep branches of radial nerve; HT, humeral trochlea; HhPT, humeral head of pronator teres muscle; MN, median nerve; red arrows, long axis view of EBA; SL-EBA, superficial layer of EBA; ML-EBA, middle layer of EBA; DL-EBA, deep layer of EBA.

The transition from muscle to tendon in the BBm necessitates a deeper understanding of the aponeurotic structures that facilitate this complex anatomical progression. To elucidate this complexity, we adopt the nomenclature proposed by Blasi et al.,^9^ which differentiates the internal bicipital aponeurosis (IBA) and the external bicipital aponeurosis (EBA) as key components of the distal biceps brachii tendinous complex (DBBTC) (Figure 1(a)). The IBA, positioned centrally within the BBm and proximal to the elbow joint level, forms an intramuscular aponeurosis that organises the parallel muscle fibres and initiates the tendinous complex of DBBT. This arrangement illustrates a sophisticated morphological progression, allowing the muscle to transition seamlessly into its tendinous components, including contributions to both the DBBT and the EBA.

The EBA, commonly referred to as the bicipital aponeurosis or lacertus fibrosus, reveals additional structural complexity, originating from the medial extensions of the IBA.^9^ As its layers extend distally and superficial to the brachial artery and median nerve, it establishes tight fascial connections with the ulnar flexor muscles, enhancing the EBA's role in stabilising the forearm’s musculature. This structural integration with the antebrachial fascia optimises the BBm leverage during supination, facilitating effective force transmission and enhancing forearm and grip functionality.^12^

### Distal tendon anatomy

Following the formation of the IBA, the BBm progresses distally into the DBBT, as it approaches its insertion at the radial tuberosity. Anatomical studies have demonstrated that the DBBT exhibits a range of insertional patterns,^12,13^ challenging the traditional view that the BBm merges into a single tendon.^14^ Recent findings reveal that the long-heads brachii tendon (LHBT) and long-heads brachii tendon (SHBT) follow distinct paths as they approach the insertional footprint. Initially positioned side by side the SHBT and LHBT undergo approximately 90° of external rotation as the tendons descend, positioning the SHBT superficial to the LHBT. Functionally, this arrangement supports the LHBT’s primary role in forearm supination, while the SHBT contributes more to elbow flexion.^12,13^

### Radial tuberosity: footprint morphology

As the DBBT approaches its insertion into the radial tuberosity, understanding the morphology of this bony landmark becomes essential to establish a baseline expectation of normal anatomy during scanning. The work by Van den Bekerom et al.^14^ provided an in-depth classification of the tuberosity’s morphology. Three types were identified: the bifid ridged type which features two prominent ridges with a trough in between; the smooth type which lacks ridges and presenting a uniform attachment surface; and the single ridged type, which is further divided into small, medium, and large subtypes based on qualitative appearance. The authors suggested that the radial tuberosity functions as a mechanical pulley, enhancing the musculotendinous unit’s leverage during forearm movements. More recent findings though,^2^ simplify this understanding into two broader categories: a narrow half-moon along the ulnar edge of the tuberosity, classified as type I; and a broader oval positioned posteromedially, classified as type II.

Awareness of the radial tuberosity's morphology is essential to establish a baseline for normal anatomical variations during sonographic evaluation. While literature extensively categorises the tuberosity into different types, it does not address potential distinctions in the bone surface morphology at the insertional sites of the LHBT and SHBT. Practical observations, however, indicate that the osseous surface at the LHBT insertion appears more oval, while the SHBT insertion appears flatter.

## Sonographic appearance of the normal distal biceps brachii tendinous complex

On ultrasound, the IBA and EBA are distinguishable structures. The IBA appears as a central hyperechoic band within the BBm, running along the muscle fibres and progressively forming the DBBT (Figure 1(b)). The EBA, originating medially from the IBA, exhibits a layered composition that is triangular to rectangular in cross-section, with variable echogenicity (Figure 1(c)).

The SHBT and LHBT, in the short-axis view, typically appear as distinct, hyperechoic structures, exhibiting the bright appearance characteristic of healthy tendons. They are situated superficial to the brachialis muscle and positioned just lateral to the brachial artery, providing a reliable landmark for sonographic identification (Figure 1(d)). More specifically, the SHBT is positioned closer to the brachial artery, appearing smaller and more flattened, while the LHBT, located lateral to the SHBT, appears slightly larger and more oval-shaped.^15^ As the scan progresses distally, the tendons exhibit a 90° rotation,^12,13^ causing the SHBT to slide superficial to the LHBT, a detail best captured in short-axis views (Figure 1(e)). At the same level, the EBA can be appreciated through its three layers – the superficial, middle, and deep – which can be differentiated in the long-axis view. The superficial and deep layers are primarily hyperechoic, while the middle layer appears hypoechoic, merging distally with the fascia of the ulnar flexor muscles (Figure 1(f)).^7,16^

In the long-axis view, the SHBT and LHBT retain their hyperechoic appearance, with a distinct fibrillar architecture.^1,5^ At the insertional footprint on the radial tuberosity, the LHBT attaches onto the larger, proximal part of the tuberosity and displays a subtle convexity along its superficial layer. This creates a slightly raised contour rather than a flat appearance, which can aid in distinguishing it from the SHBT. In addition, fibres of the LHBT can be appreciated extending into the proximal and deeper aspects of the tuberosity. In contrast, the SHBT attaches solely to the distal and superficial areas of the radial tuberosity that extends further distally, occupying only the uppermost part of the insertional footprint. It maintains a consistently flat and straight appearance along its superficial layer, providing a clear visual distinction from the LHBT^14,17^

While literature does not explicitly document differences in the osseous surface morphology of the radial tuberosity between the two tendinous insertions, practice-based observations indicate that, on ultrasound, the bone surface at the LHBT insertion appears more oval, whereas the surface at the SHBT insertion appears flatter.

A summary of proposed key sonographic characteristics distinguishing the LHBT and SHBT in the long-axis view is provided in Table 1, incorporating documented features and practice-based insights to guide ultrasound assessment.

**Table 1.** Proposed sonographic characteristics distinguishing the long head and short head of the distal biceps brachii tendon in the long-axis view.

Sonographic feature	LHBT	SHBT
Superficial layer appearance	Subtle convexity, creating a raised contour	Flat and straight
Insertional attachment	Extends into the proximal and deeper aspects of the radial tuberosity	Attaches to the distal and superficial areas, occupying the uppermost part of the tuberosity
Radial tuberosity osseous morphology	Oval-shaped bone surface at insertion site	Flatter bone surface at insertion site

## The Radial arc technique

This section introduces the Radial arc technique, a didactic and systematic ultrasound scanning technique from a medial approach designed to facilitate the identification of the DBTT insertion. The term Radial arc is derived from the characteristic arc-like arrangement of the radial head, neck and tuberosity, observed during the scanning process in the fourth step of our five-step approach, where the radial head is visualised as a hill on one side, the radial neck as a valley in the centre, and the radial tuberosity as another hill on the other side, collectively resembling an arc. This nomenclature not only captures the essence of the anatomical landmarks’ configuration but also aids in visualising the dynamic relationships among these structures. The Radial arc technique aims to simplify learning and standardise the medial ultrasound approach for diagnosing DBBT-related conditions, including tendinosis, tears, and bicipitoradial bursa pathologies.

Ultrasound images were captured using an ML6–15 MHz linear transducer. In the long-axis view, the probe marker was oriented cephalically, towards the head. In the short-axis view, the probe marker was directed towards the midline of the body. Consequently, in long-axis views, the left side of the sonograms consistently correspond to the proximal end, and the right side to the distal end of the anatomical structures being visualised; while in short-axis views, the left side represents the medial aspect and the right side the lateral aspect, respectively.

### Patient positioning

**Figure 2.**
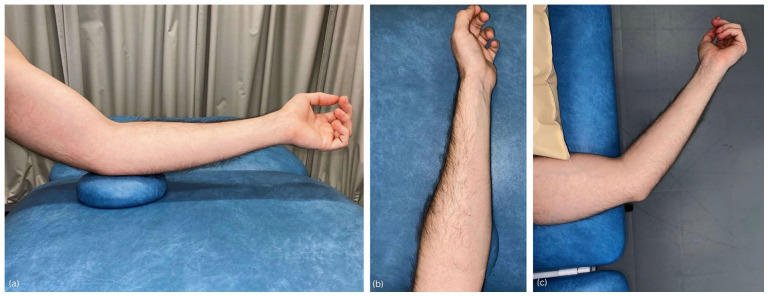
Patient positioning. (a) Patient seated facing the clinician, left elbow on cushion at 30° flexion for optimal probe access and distal biceps brachii tendon visualisation. (b) Left forearm in a near-neutral position, close to halfway between pronation and supination. (c) Patient lying on side with the left arm slightly off the plinth and the distal humerus supported for comfort and accessibility.

The patient is positioned seated on a chair facing the clinician, with the elbow comfortably rested on a small cushion to facilitate stability and ease of access (Figure 2(a)). This arrangement permits convenient manipulation of the clinician’s hand and the ultrasound probe along the medial aspect of the elbow and forearm. The elbow is maintained at a flexion angle of 30°, which positions the DBBT in a taut state. The forearm is kept in a near-neutral position, close to halfway between pronation and supination; this aligns the radial tuberosity in a way that can be clearly visualised during the scanning technique. This neutral forearm position is achieved by instructing the patient to fully supinate the forearm initially and then relax it back to a natural midpoint (Figure 2(b)). In addition, the Radial arc technique can also be performed with the patient lying on their side; the examined arm should be slightly off the plinth with the distal humerus supported by the plinth, providing an alternative positioning option that caters to patient comfort and different clinical settings (Figure 2(c)).

### Five-step approach to long-axis imaging of the DBBT using medial approach

#### Step 1

##### Probe orientation

**Figure 3.**
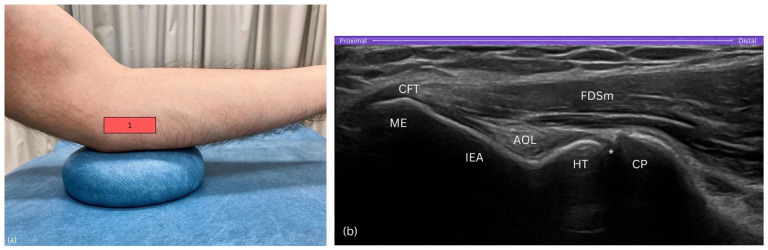
Step 1. (a) Probe on medial epicondyle, aligned horizontally. (b) View includes the medial epicondyle (ME), humeral trochlea (HT), and coronoid process (CP), excluding ulnar shaft. AOL, anterior oblique ligament of the medial collateral ligamentous complex; CP, coronoid process; CFT, common flexor tendon; FDSm, flexor digitorum superficialis muscle; HT, humeral trochlea; IEA, inferior epicondylar aspect; ME, medial epicondyle; asterisk (*), humero-ulnar joint.

Position the proximal end of the probe on the medial epicondyle, ensuring the distal end is aligned horizontally, independent of the ulna's longitudinal axis (Figure 3(a)). With the elbow maintained at 30° of flexion, this orientation facilitates an optimal sonographic depiction of the medial epicondyle, inferior epicondylar aspect, humeral trochlea, and the coronoid process of the ulna, while excluding the ulna shaft from the visual field. This setup also optimally displays the common flexor tendon (CFT) and medial collateral ligament (MCL) in a long axis view (Figure 3(b)).

##### Purpose

This initial positioning of the probe establishes the medial epicondyle as a reference point, allowing visualisation of key medial elbow structures before progressing distally.

#### Step 2

##### Probe orientation

**Figure 4.**
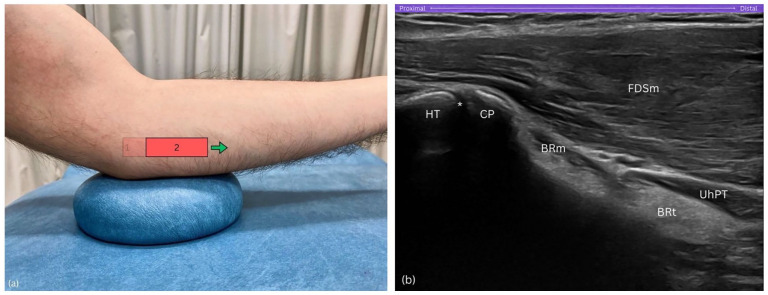
Step 2. (a) Distal slide of the probe in a true horizontal direction. (b) Medial epicondyle (ME) and inferior epicondylar aspect (IEA) exit sonogram, while the humeral trochlea (HT) and coronoid process (CP) are now situated on the left side of the sonogram. BRm, brachialis muscle; BRt, brachialis tendon; CP, coronoid process; FDSm, flexor digitorum superficialis muscle; HT, humeral trochlea; UhPT, ulnar head of pronator teres; asterisk (*), humero-ulnar joint.

Gradually slide the probe distally in a strictly horizontal orientation until the medial epicondyle is no longer visible on the sonogram (Figure 4(a)). This movement repositions the humeral trochlea and the coronoid process of the ulna to the left side of the sonogram (Figure 4(b)).

##### Purpose

Sliding the probe distally in this step helps transition the view from the medial epicondyle to the coronoid process, reorienting key landmarks on the sonogram.

#### Step 3

##### Probe orientation

**Figure 5.**
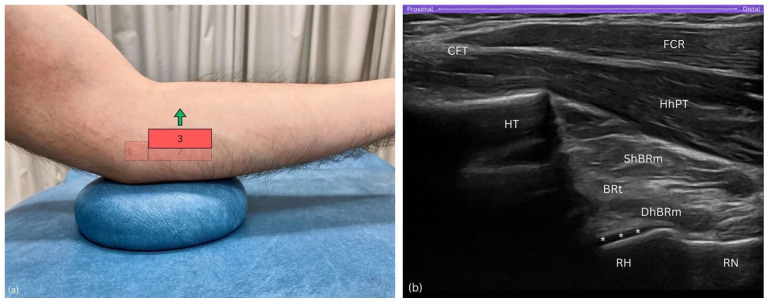
Step 3. (a) Anterior sweep of the probe towards forearm's midline. (b) The radial head (RH) appears with hyperechoic signal, while the humeral trochlea (HT), resembling a cliff, remains visible. BRt, brachialis tendon; CFT, common flexor tendon; DhBRm, deep head of brachialis muscle; FCR, flexor carpi radialis; HT, humeral trochlea; HhPT; humeral head of pronator teres; RH, radial head; RN, radial neck; ShBRt, superficial head of brachialis muscle; asterisks (*), hyaline cartilage.

Sweep the probe anteriorly, towards the forearm's midline (Figure 5(a)). As you sweep anteriorly and the coronoid process fades from view, the radial head emerges, producing a distinct hyperechoic signal when the probe achieves a perpendicular angle to the bone. This critical alignment is verified by the visual confirmation of anechoic hyaline cartilage covering the radial head. Throughout this manoeuvre, maintain the humeral trochlea, resembling a cliff, within the left visual field of the sonogram (Figure 5(b)).

##### Purpose

Sweeping the probe anteriorly towards the forearm’s midline is essential for locating the radial head, a key component of the Radial arc technique. A pitfall at this step is neglecting to increase the depth setting on the ultrasound machine. Even with correct probe orientation, failure to adjust the depth may result in missing the radial head entirely, as it is located deeper within the sonographic field; a factor that varies depending on the thickness of subcutaneous adipose tissue and muscle tissue layers.

#### Step 4

##### Probe orientation

**Figure 6.**
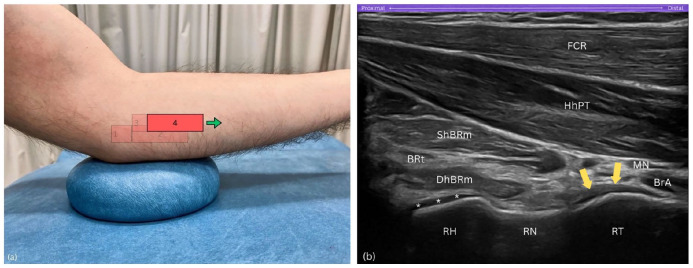
Step 4. (a) Further distal slide of the probe in a true horizontal direction. (b) Radial arc: view includes the radial head (RH) as a hill on the left, radial neck (RN) as a valley in the centre, and radial tuberosity (RT) as a hill on the right side of the sonogram. BRt, brachialis tendon; BrA, brachial artery; DhBRm, deep head of brachialis muscle; FCR, flexor carpi radialis; HhPT; humeral head of pronator teres; MN, median nerve; RH, radial head; RN, radial neck; RT, radial tuberosity; ShBRt, superficial head of brachialis muscle; asterisks (*), hyaline cartilage; yellow arrows, distal biceps brachii tendon.

Once optimal visualisation is achieved, with the hyperechoic cortex of the radial head distinctly outlined along with the surrounding anechoic hyaline cartilage, continue sliding the probe further distally in a true horizontal direction (Figure 6(a)). This manoeuvre should align the radial head on the left side of the sonogram resembling a hill, the radial neck in the centre like a gentle slope resembling a valley, and the radial tuberosity on the right side appearing as another hill – effectively displaying the sequential anatomical landmarks across the sonogram (Figure 6(b)).

##### Purpose

Sliding the probe further distally along the Radial arc reveals the sequential landmarks of the radial head, neck, and tuberosity, creating a continuous arc-like appearance.

#### Step 5

##### Probe orientation

**Figure 7.**
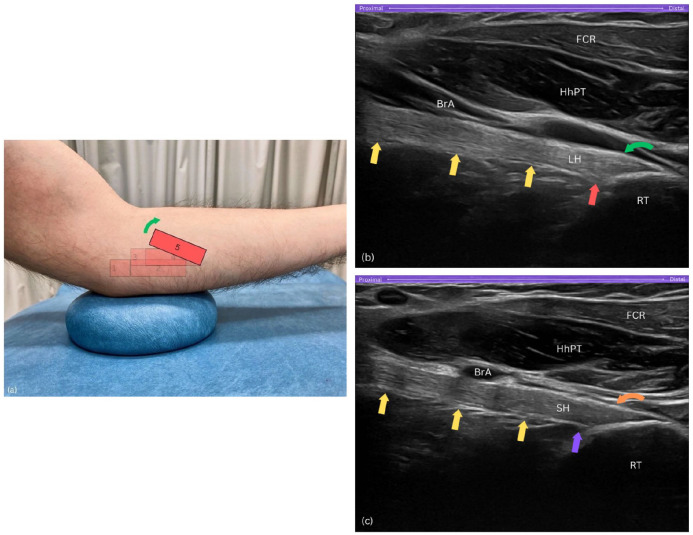
Step 5. (a) The proximal end of the probe is upwardly rotated by about 15°. (b) Long-axis view of the long head of biceps brachii tendon (LH) insertion showing a subtle convexity – raised contour – along its superficial layer, with insertional fibres extending proximally and deeply into the radial tuberosity (RT). (c) Long-axis view of the short head of biceps tendon (SH) insertion displaying a flat, straight superficial layer, with fibres confined to the uppermost, distal part of the footprint. BrA, brachial artery; FCR, flexor carpi radialis; HhPT, humeral head of pronator teres; RT, radial tuberosity; yellow arrows, distal biceps brachii tendon; LH, long head of biceps tendon; red arrow, proximal and deep insertional fibres of LH on tuberosity; curved green arrow, raised contour of LH’s superficial layer; SH, short head of biceps tendon; purple arrow, SH footprint lacks proximal fibres; curved orange arrow, flat and straight superficial layer of SH.

Ensure the radial tuberosity remains on the right side of the sonogram (Figure 7(a)). Then, keep the distal end of the probe stable while pivoting the proximal end of the probe upwards by about 15 degrees. This manoeuvre aligns the DBBT in its long axis view, through the pronator teres window (Figure 7(b) and (c)).

To distinguish the SHBT and LHBT at the insertional footprint, a subtle sliding manoeuvre can be performed. To visualise the LHBT, the probe is subtly slid posteriorly, where the LHBT displays an oval-shaped attachment on the larger, proximal part of the tuberosity with a slight convexity along its superficial layer, creating a raised contour appearance (Figure 7(b)). In contrast, to visualise the SHBT, the probe is gently slid anteriorly, where the SHBT presents as a fan-shaped structure attaching to the distal portion of the radial tuberosity, occupying the uppermost and superficial areas, and exhibiting a flat and straight appearance along its superficial layer (Figure 7(c)). This subtle manoeuvring allows for clear differentiation between the two tendons at their distinct attachment sites. At this location, dynamic assessment of both tendinous heads can be performed by rotating the forearm between pronation and supination.

##### Purpose

Pivoting the probe at the end facilitates alignment with the DBBT’s long axis, allowing both static and dynamic assessments through the pronator teres window, as well as identification of the two distinct heads of the DBBT.

## Conclusion

The Radial arc technique provides a structured, five-step sonographic approach to visualising the DBBT in long axis through a medial approach. This technique aims to simplify the often-complex sonographic examination of the DBBT, enhancing diagnostic accuracy for conditions such as ruptures, tendinopathic changes, bicipitoradial bursitis, and space-occupying lesions, which are critical for guiding patient management. By standardising this approach, the Radial arc technique seeks to support clinicians across varied experience levels, from students and early-career practitioners to experienced sonographers, making MSK ultrasound more accessible and consistent across clinical and educational settings. Further studies are encouraged to validate the reproducibility and diagnostic utility of this technique in diverse clinical settings and among different practitioners and operators, crucial for its adoption in sonographic diagnostics.
